# Mesosalpinx hernia of the sigmoid colon: A cause of lower abdominal pain in women

**DOI:** 10.1002/ccr3.2754

**Published:** 2020-02-26

**Authors:** Taiki Higaki, Seigo Urushidani, Akira Kuriyama, Tetsunori Ikegami

**Affiliations:** ^1^ Emergency and Critical Care Center Kurashiki Central Hospital Kurashiki Okayama Japan

**Keywords:** acute abdomen, adnexa, colonoscopy, hernia

## Abstract

Diseases of the uterus and adnexa uteri should be considered when evaluating female patients with lower abdominal pain. Diseases caused by defects in the supporting ligaments of the female reproductive system should also be considered.

## Graphical abstract

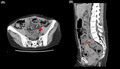

## 1 INTRODUCTION

A 38‐year‐old woman presented with acute lower abdominal pain. An emergency laparotomy revealed an internal hernia of the sigmoid colon through a left mesosalpinx.

Diseases of the uterus and adnexa uteri are generally considered among the diagnostic possibilities when evaluating female patients with acute lower abdominal pain in emergency department (ED) settings. However, diseases caused by defects in the supporting ligaments of the female reproductive system may also cause lower abdominal pain. Here, we present a case of a woman with an internal hernia of the sigmoid colon that occurred through a mesosalpinx defect.

## 2 CASE REPORT

A 38‐year‐old woman presented to our hospital with continuous acute abdominal pain, rated as a severity of 10/10 on the numerical rating scale. She reported several episodes of loose stools, but no hematochezia, nausea, or vomiting. The pain was located centrally in the lower abdomen and had not moved or spread. The patient reported an irregular menstrual cycle. Her last menstrual period had started 10 days prior and had lasted for 7 days. She did not experience atypical genital bleeding or hematuria with back pain. She had a medical history of caesarean section and untreated uterine fibroids, but denied any current gynecological diseases or inflammatory bowel diseases.

**Figure 1:**
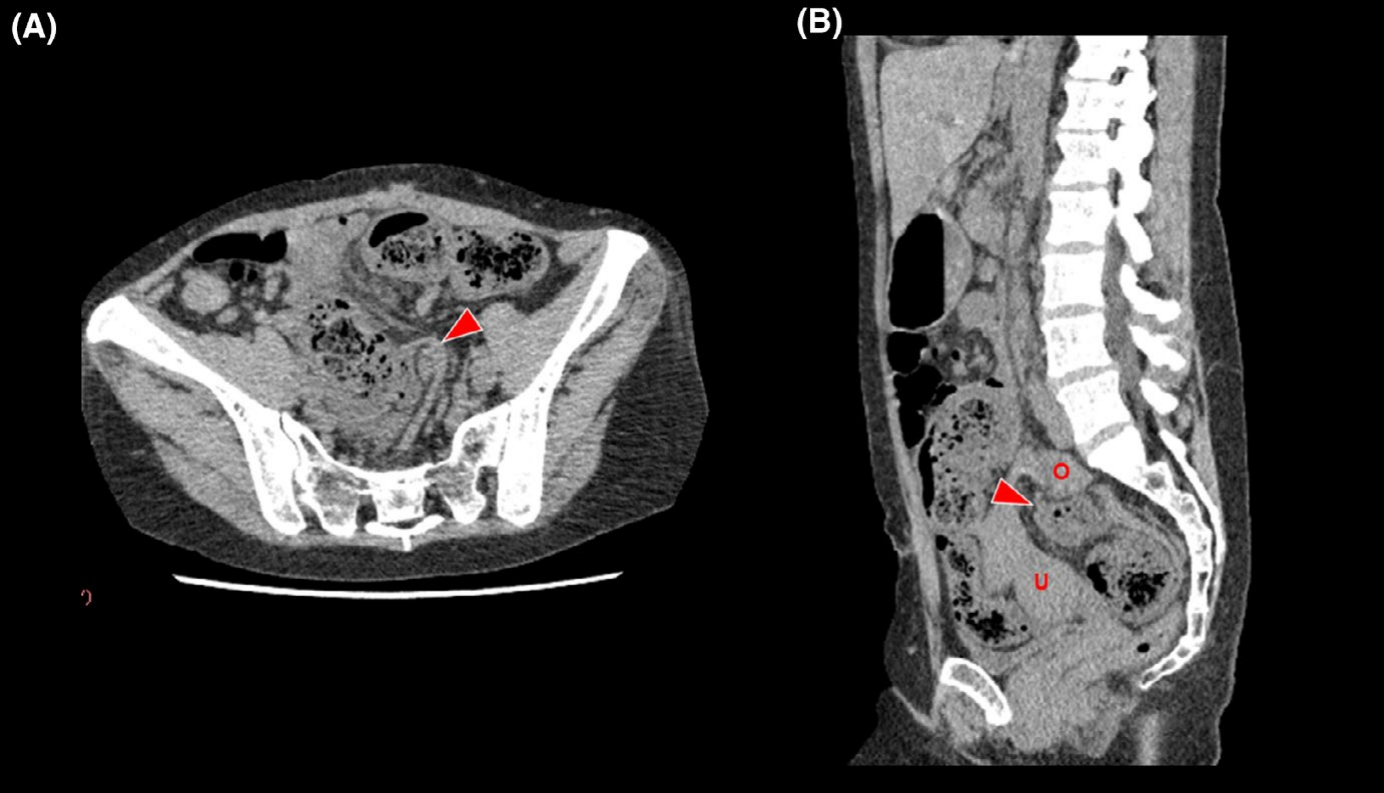
A, An axial view of a computed tomography (CT) scan showed stenosis of the sigmoid colon (arrowhead). B, A sagittal view of the CT scan showed the protrusion of the sigmoid colon (arrowhead) between the uterus (U) and ovary (O)

Her vital signs were as follows: blood pressure, 120/69 mm Hg; heart rate, 100 beats/min; respiratory rate, 19 breaths/min; SpO_2_, 100% with room air; body temperature, 37.2°C; and Glasgow Coma Scale score, E4V5M6. A physical examination revealed tenderness in her middle lower abdomen without muscular guarding. Laboratory investigations revealed a white blood cell count of 7200/mm^3^. A pregnancy test was negative. Abdominal ultrasonography (US) did not indicate pyelectasis or a mass lesion in her lower abdomen. Abdominal computed tomography (CT) performed to evaluate the cause of the abdominal pain revealed stenosis of the sigmoid colon with dilation on the oral side, with no abdominal free air, significant ascites, or colon diverticula (Figure 1). We performed a colonoscopy to evaluate the cause of the sigmoid colon stenosis, which revealed a narrow stenotic lesion and discoloration of the intestinal mucosa. We therefore suspected mesenteric ischemia.

An emergency laparotomy was performed, which revealed strangulation of the sigmoid colon by a fallopian tube, ovarian suspensory ligament, and ovarian ligament. The patient was diagnosed with an internal hernia of the sigmoid colon through a defect in the left mesosalpinx. The strangulated colon was edematous, and there was a small amount of ascites in the abdominal cavity. The herniated colon was not necrotic and was released from ligamental strangulation. Her postoperative recovery was uneventful, and she was discharged on the fourth day after surgery.

## 3 DISCUSSION

Evaluations of patients with acute abdominal pain in the ED generally consider common diseases and acute abdominal or vascular diseases, such as aortic dissection or mesenteric ischemia.^1^ For female patients, sex‐specific diseases such as ectopic pregnancy, a ruptured ovarian cyst, ovarian torsion, or pelvic inflammatory disease should also be considered.^2^ Although our patient presented with acute lower abdominal pain, we could not narrow the differential diagnosis based on her history or the findings from a physical examination and US. The CT results suggested a potential malignancy or sigmoid volvulus, leading us to perform a colonoscopy.^3^, ^4^ However, an emergency laparotomy revealed the actual diagnosis.

Internal hernias involve a protrusion of the viscera through the peritoneum or mesentery and into a compartment of the abdominal cavity.^5^ Pelvic or supravesical hernias comprise 6% of all internal hernias.^5^ Kamata et al reported that the etiologies of internal hernias through the mesosalpinx included congenital factors, pregnancy, delivery, adhesion due to pelvic inflammatory disease, distortion, reduced elasticity of ligaments, and thinning of the mesosalpinx due to an ovarian tumor.^6^ Although our patient had a history of caesarean section, the cause of the hernia in this case remains unknown.^7^

We have identified five previous case reports describing internal hernias of the intestine through the mesosalpinx (Table 1).^6^, ^7^, ^8^, ^9^, ^10^ All patients were older than 40 years, and all but one complained of abdominal pain, nausea, and vomiting. One was a grand multipara, and two had a history of abdominal surgery. In all five cases, the small intestine was obstructed, and the diagnoses were made through laparotomy. However, to the best of our knowledge, an internal hernia of the sigmoid colon through the mesosalpinx, as in our case, has not been previously reported.

**Table 1:** Reported cases of mesosalpinx hernia

Author (y)	Age (y)	Symptoms	Medical history	Intestinal ischemia
Kamata (1989)^6^	72	Nausea, vomiting, upper abdominal pain	Not reported	No
Tan (2010)^7^	65	Vomiting, abdominal pain, constipation, distention	Appendicitis	Yes
Petereit (1973) 8	57	Nausea, vomiting, abdominal pain	Not reported	No
Dunn (1926)^9^	44	Lower abdominal pain	Grand multipara	No
Garcia‐Oria (2007)^10^	43	Vomiting, right upper abdominal pain	Bowel surgery	No

In four of the five reported cases, surgery was performed because abdominal radiography suggested an intestinal obstruction. In one case, small bowel resection was performed because of mesenteric ischemia. In our case, CT enabled a diagnosis of mesenteric obstruction, and colonoscopy revealed impaired blood flow to the intestine. These findings enabled us to perform an emergency laparotomy.

## 4 CONCLUSION

Diseases of the uterus and adnexa uteri should be considered when evaluating female patients with lower abdominal pain. In such cases, diseases caused by defects in the supporting ligaments of the female reproductive system should also be considered, especially since such conditions need emergency laparotomy.

## CONFLICT OF INTEREST

None.

## AUTHOR CONTRIBUTIONS

TH: is the first author, managed the patient in the emergency department, and drafted the manuscript. SU: reviewed and edited the manuscript, prepared the manuscript for the submission, and communicated with editors. AK: reviewed and edited the manuscript and supervised the writing and submitting process. All authors discussed and approved the submitted manuscript.
